# Frequency of T-Cell Progenitors in Nude Mice

**DOI:** 10.1155/1995/89597

**Published:** 1995

**Authors:** I. Nicholas Crispe

**Affiliations:** Immunobiology Section Yale Medical School 310 Cedar Street New Haven Connecticut 06510 USA

**Keywords:** CFU-S, CFU-T, nude mice, prothymocytes, stem cells

## Abstract

The hypothesis that prothymocytes are distinct from and regulated independently of multilineage hemopoietic progenitors was tested by enumeration of these two cell populations in normal versus congenitally athymic (nude) mice. The absence of a thymus and of peripheral T cells in nude mice had no effect on the frequency of either multilineage progenitors (day 12 CFU-S) or prothymocytes (CFU-T), suggesting that there is no feedback regulation of CFU-T frequency. Thymus seeding from the bone marrow is therefore likely to be regulated by the availability of niches for prothymocyte maturation, rather than by feedback control of prothymocyte production.


*Developmental Immunology*, 1996, Vol 4, pp. 257–261
Reprints available directly from the publisher
Photocopying permitted by license only

© 1996 OPA (Overseas Publishers Association)
Amsterdam B.V. Published in The Netherlands
by Harwood Academic Publishers GmbH
Printed in Singapore

### Frequency of T-Cell Progenitors in Nude Mice

I. NICHOLAS CRISPE

*Immunobiology Section, Yale Medical School, 310 Cedar Street, New Haven, Connecticut 06510*

The hypothesis that prothymocytes are distinct from and regulated independently of multilineage hemopoietic progenitors was tested by enumeration of these two cell populations in normal versus congenitally athymic (nude) mice. The absence of a thymus and of peripheral T cells in nude mice had no effect on the frequency of either multilineage progenitors (day 12 CFU-S) or prothymocytes (CFU-T), suggesting that there is no feedback regulation of CFU-T frequency. Thymus seeding from the bone marrow is therefore likely to be regulated by the availability of niches for prothymocyte maturation, rather than by feedback control of prothymocyte production.

KEYWORDS: CFU-S, CFU-T, nude mice, prothymocytes, stem cells.

### INTRODUCTION

Early T-lineage cells identified in the thymus differ from hemopoietic stem cells in their surface phenotype, differentiation potential, and burst size in intrathymic adoptive transfer (Scollay et al., 1988; Wu et al., 1991). At first sight, these observations support the idea that T-lineage progenitors originate in the bone marrow as one of the differentiation products of multilineage stem cells and migrate to the thymus where they find a microenvironment that supports their further differentiation.

If this model is true, progenitor thymocytes are distinct from hemopoietic stem cells and may be separable from them, either by physical methods or by using specific assays. A major problem in the clarification of the relationship between stem cells and T-cell progenitors is that very early T cells may only be detected by their ability to repopulate the thymus either in fetal organ culture or in vivo, assays in which highly purified stem cells are also active (Kingston et al., 1985; Spangrude et al., 1988).

Donor-type bone-marrow–derived cells proliferate very rapidly in irradiated host thymi, suggesting that the production of thymocytes is subject to some form of feedback regulation (Hirokawa et al., 1985). However, the level at which such feedback regulation might occur is unknown. If T-cell progenitors are indeed distinct from stems cells, regulation of the T-cell supply may occur at the level of the production of these progenitors. The present study aims to test this hypothesis.

Nude mice exhibit a developmental defect in which the ectodermal and endodermal components of the thymus fail to unit. In these animals, the thymus rudiment is not colonized by progenitor cells, and very few mature circulating T cells are produced (Cordier and Heremans, 1975). The few T cells that are seen as nude mice age may be escapees from extrathymic sites of T-cell production, such as the intestinal epithelium, which do not normally contribute significantly to the circulating T-cell pool (Kennedy et al., 1992; Ohteki et al., 1992). If T-cell progenitors are subject to feedback regulation, the lack of T cells in nude mice should result in their overproduction. Even if T-cell progenitors are produced in normal numbers in nude mice, the lack of a "sink" for such cells would be expected to cause their accumulation, either in the bone marrow or in the periphery.

To test these ideas, nude versus normal bone marrow was tested for multilineage progenitor cells (day 12 CFU-S) as an index of overall hemopoietic progenitor production, and for the frequency of T-cell progenitor cells (CFU-T) in a limiting dilution intrathymic adoptive transfer assay (Spangrude et al., 1988). The frequencies of CFU-S and CFU-T in bone marrow, and of CFU-T in the spleen, were identical in B6 and B6-nude mice. This argues

* Corresponding author.

against the model that T-cell progenitors are a subset of cells distinct from multilineage stem cells and subject to feedback regulation by the peripheral T-cell pool. The possible alternative models are discussed.

### RESULTS AND DISCUSSION

The bone marrow of B6 and B6-nude mice contained identical numbers of CFU-S, within the limits of the assay (Fig. 1). The frequency of CFU-S was 1 in 6993 in B6 and 1 in 6186 in B6-nude. Thus, nude mice were not responding to their T-cell deficiency with an overproduction of multilineage progenitor cells. This is not surprising, because the cell population assayed as day 12 CFU-S can give rise to all hemopoietic lineages in short-term repopulation and radioprotection assays, although these cells are distinct from self-renewing, long-term, repopulating stem cells (Jones et al., 1990). If T-cell progenitors were indeed increased in the nude mouse, the expanded population would be expected to be at a post-CFU-S differentiation stage.

To look for an expanded population of bone-marrow T-cell progenitors, nude versus normal bone marrow as injected intrathymically in limiting dilution into Thy-1 congenic host mice. Analysis of the frequencies of 'nonrepopulated thymus lobes showed that there was no excess of pre-T cells in the nude bone marrow (Fig. 2). The estimated frequencies of CFU-T were 1 in 41,630 in B6 and 1 in 37,653 in B6-nude. Surprisingly, CFU-T from both normal and nude mice were less frequent than CFU-S by about sixfold. Highly enriched bone-marrow stem cells have been shown to score with an efficiency of 1 in 5 in the CFU-T assay and 1 in 10 in the CFU-S assay (Spangrude et al., 1988). The present data therefore suggest that bone-marrow CFU-S are more frequent than CFU-T.

At this stage, several possibilities remain: The CFU-T assay detects only true stem cells, while the day 12 CFU-S assay also detects more differentiated cells; or the CFU-T assay detects an independent subset or progenitor T cells that are not subject to feedback regulation by existing T cells; or a regulated population of progenitor T cells exists but it is not found in the bone marrow, possibly because these cells are programmed to leave as they undergo lineage commitment.

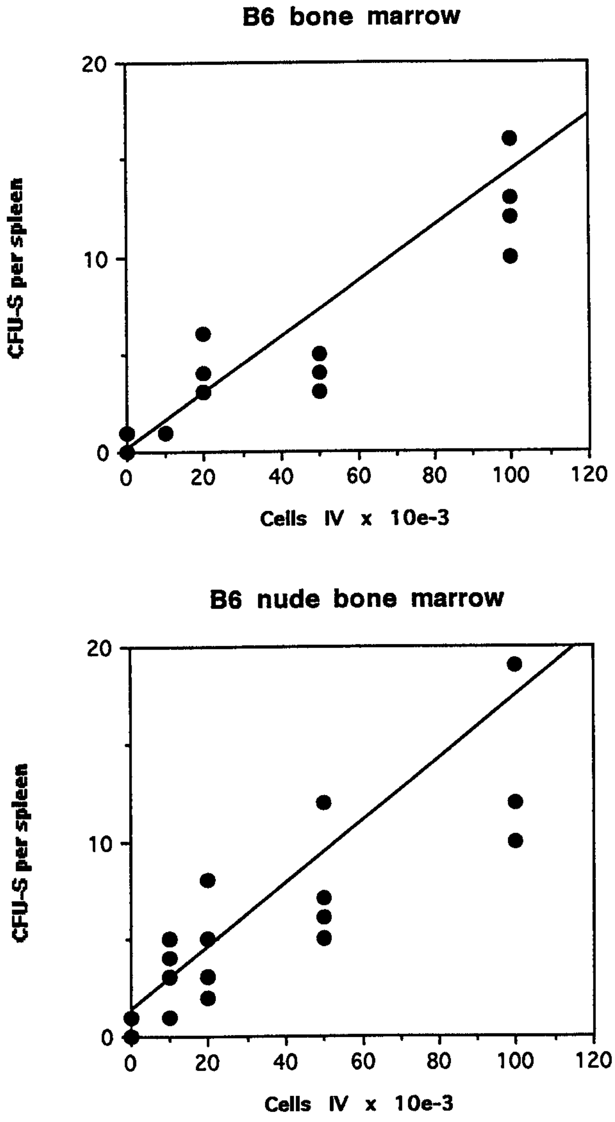

FIGURE 1 Assay of B6 and B6-nude bone marrow cells for day 12 CFU-S. Each data point represents one host animal. The frequency of CFU-S was estimated at 1 in 6186 cells for B6-nude and 1 in 6993 for B6. Thus, multilineage progenitor-cell frequencies are unaffected by the lack of T cells in nude mice.

Previous studies have shown that some intrathymic progenitor activity can be isolated from spleen (Goldschneider et al., 1986). Accordingly, T-depleted B6 spleen cells were compared to similarly treated B6-nude spleen cells in the CFU-T assay (Fig. 3). The estimated frequencies of splenic CFU-T were identical; 1 in 153,756 in B6 and 1 in 159,913 in B6-nude.

Two conclusions may be drawn from these studies. First, the ratio between CFU-S and CFU-T is not

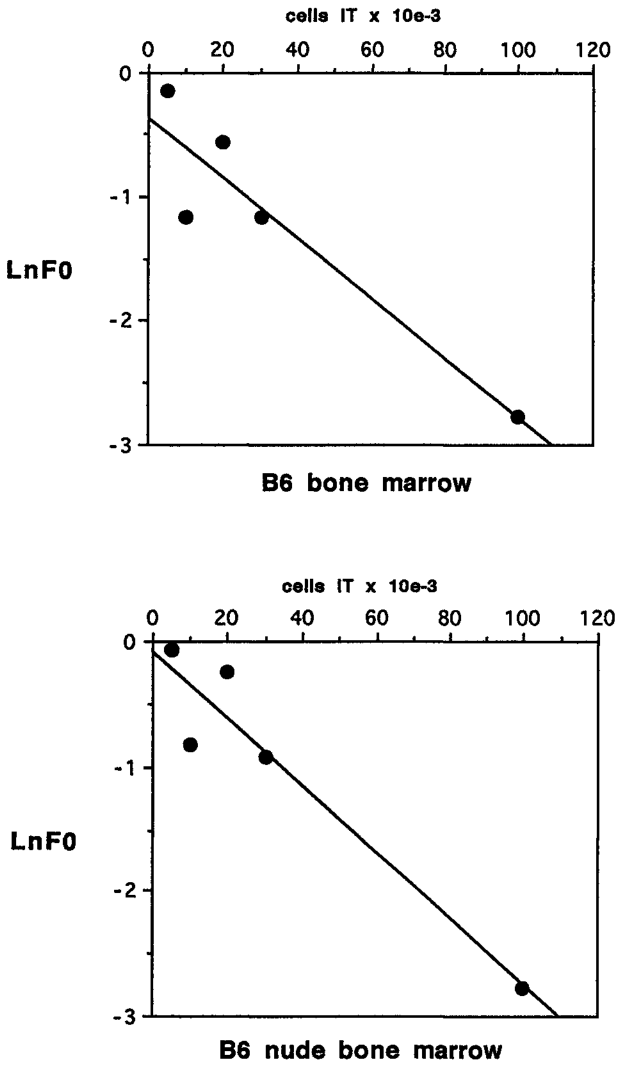

FIGURE 2 Frequencies of T-cell progenitors (CFU-T) in B6 and B6-nude bone marrow. Unfractionated bone-marrow cells were injected intrathymically in limiting dilution into Thy-1 congenic hosts. Data points represent the frequency of negative thymus lobes out of a group of 14–18 lobes (in 7–9 animals). The CFU-T frequencies estimated were 1 in 37,653 for B6-nude and 1 in 41,630 for B6. Thus, the lack of T cells in nude mice did not increase the bone-marrow CFU-T frequency. LnF0: natural log of the frequency of negative thymus lobes.

perturbed in nude mice. This, plus the observation that highly purified bone-marrow stem cells are active in the CFU-T assay (Spangrude et al., 1988), is compatible with the proposition that bone-marrow prothymocytes are indistinguishable from stem cells. However, we have no direct evidence that the same cells are being detected in the CFU-S and CFU-T assays. Second, the fact that the ratio between bone-marrow prothymocytes and splenic prothymocytes is unchanged in nude mice suggests the rate of discharge of bone-marrow prothymocytes into the periphery is unaffected by end-product (T-cell) feedback.

Until a population of prethymic, T-lineage-committed prothymocytes is clearly identified, the most plausible hypothesis is that no such cell population exists. Instead, the thymus is probably seeded by multilineage progenitor cells (essentially, stem cells) that undergo lineage commitment after they enter the thymus. This position is difficult to reconcile with the analysis of the differentiation potential of the earliest thymocytes so far characterized, the CD4-low, CD44-high progenitor described by Wu and colleages (Wu et al., 1991). This cell population was competent to differentiate into T cells, B cells, and dendritic cells (Ardavin et al., 1993), while purified stem cells injected into an irradiated thymus also give rise to granulocytes (Spangrude and Scollay, 1990). A search for non-T-differentiation potential in CD4–CD8– or CD3–CD8– thymocytes, using the CFU-S and IL-3-dependent CFU-C assays, revealed less than one CFU-S and less than one CFU-C per thymus (I.N.C., unpublished studies), To reconcile the present experiments with these data, it is necessary to propose that stem cells that arrive in the thymus very rapidly undergo lineage commitment. It may be that in the normal thymic microenvironment, such commitment is mandatory for their survival.

### MATERIALS AND METHODS

### Animals

C57BL/6 (B6) mice were obtained from the Jackson Laboratory (Bar Harbor, ME); C57BL/6-nu/nu (B6 nude) mice were from Taconic (Germantown, NY); and B6-PL-Thy-1$^{a}$ mice were bred in the Immunobiology Animal Unit at Yale Medical School. All animals were maintained under SPF conditions.

### Precursor Cell Populations

Bone-marrow cells were isolated from the femora of B6 or B6-nude mice. Single-cell suspensions were prepared in Hanks Balanced Salt Solution (HBSS), counted, and cell concentration was adjusted to allow the injection of different numbers of cells in a constant volume. Spleen cells were depleted of

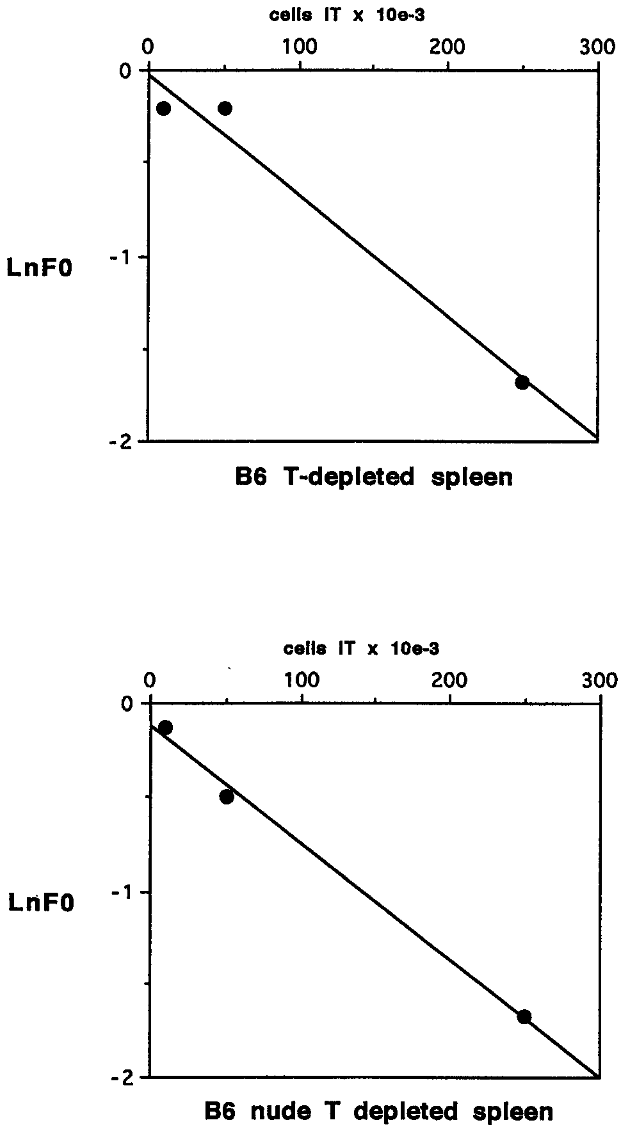

FIGURE 3 Frequencies of CFU-T in T-depleted spleen cells of B6 and B6-nude. Data are represented as in Fig. 2, and are based on groups of 16–18 lobes (8–9 animals). The CFU-T frequency estimate was 1 in 159,913 for B6 nude and 1 in 153,756 for B6. Thus, the spleen of B6-nude mice was not overloaded with unused T-cell progenitors. LnFO: natural log of the frequency of negative thymus lobes.

mature T cells by treatment with a cocktail of cytotoxic anti-CD4 and anti-CD8 monoclonal antibodies, and complement. Anti-Thy-1 antibodies were avoided because of the known expression of this marker on stem cells (Muller-Sieburg et al., 1986). The antibodies used were 3.168 (Sarmiento et al, 1980) and RL.172.4 (Ceredig et al., 1985), and cells were allowed to bind antibody for 30 min on ice. Guinea pig complement (Gibco, Grand Island, NY) was added, and the cell suspension was incubated at 37°C for 45 min. DNAse was added to prevent clumping of cell debris. Finally, viable cells were isolated by density-gradient centrifugation (Davidson and Parish, 1975).

### CFU-S Assay

Host B6 animals were irradiated with 7.5 Gy (750 rad) from an X-ray source. Between $10^4$ and $10^5$ bone-marrow cells in HBSS were injected IV, and animals were maintained on acidified water in filter frame cages thereafter. Control animals were injected with HBSS alone. Macroscopic colonies in the spleen were counted at 12 days.

### CFU-T Assay

Host B6-PL-Thy-$1^a$ animals were irradiated with 6.0 Gy (600 rad). Between $5 \times 10^3$ and $10^5$ precursor cells were injected intrathymically (IT), using the sternal split method, as previously described (Goldschneider et al., 1986; Crispe et al., 1987). Each thymus lobe was injected separately. After 21 days, a single-cell suspension was prepared from each thymus lobe, and stained with the anti-Thy-1.2 antibody 13.4 (Marshak-Rothstein et al., 1979) to detect donor-derived thymocytes. Positive lobes were typically 5–50% donor-cell (Thy-1.2) positive by FACS. Any lobe with no visible Thy-1.2 + cells (i.e., < 0.5%) was scored as negative. The frequency of thymus lobes that were not recolonized at each cell dose was used to construct limit-dilution curves. The natural log of the frequency of negative lobes (LnF0) versus the cell dose gave straight lines for both B6 and B6-nude precursor cells. Thus, a precusor frequency could be calculated.

### ACKNOWLEDGMENTS

The author wishes to thank Bernarda Strauss and Susan Welsh for technical assistance. This work was supported by a Cancer Research Institute Investigator Award.

*(Received May 17, 1995)*

*(Accepted July 17, 1995)*

### REFERENCES

Ardavin C., Wu L., Chung-Leung L., and Shortman K. (1993). Thymic dendritic cells and T cells develop simultaneously in the thymus from a common precursor population. Nature **362**: 761–763.

Ceredig R., Lowenthal J.W., Nabholz M., and MacDonald H.R. (1985). Expression of interleukin-2 receptors as a differentiation marker on intrathymic stem cells. Nature **314**: 98–100.

Cordier A.C., and Heremas J.F. (1975). Nude mouse embryo: Ectodermal nature of the primordial thymic defect. Scand. J. Immunol. **4**: 193–196.

Crispe I.N., Moore M.W., Husmann L.A., Smith L., Bevan M.J., Shimonkevitz R.P. (1987). Differentiation potential of subsets of CD4 – , CD8 – thymocytes. Nature **329**: 336–339.

Davidson W., and Parish C.R. (1975). A procedure for removing dead cells and red cells from lymphoid cell suspensions. J. Immunol. Metho. **7**: 291.

Goldschneider I., Komschlies K.L., and Greiner D.L. (1986). Studies of thymocytopoiesis in rats and mice. I. Kinetics of appearance of thymocytes using a direct intrathymic adoptive transfer assay for thymocyte precursors. J. Exp. Med. **163**: 1–17.

Hirokawa K., Sado T., Kubo S., Kamisuka H., Hitomi K., and Utsuyama M. (1985). Intrathymic T cell differentiation in radiation bone marrow chimeras and its role in T cell emigration to the spleen. An immunohistochemical study. J. Immunol. **134**: 3615–3624.

Jones R.J., Wagner J.E., Celano P., Zicha M.S., and Sharkis S.J. (1990). Separation of pluripotent haematopoietic stem cells from spleen colony-forming cells. Nature **347**: 188–189.

Kennedy J.D., Pierce C.W., and Lake J.P. (1992). Extrathymic T cell maturation. Phenotypic analysis of T cell subsets in nude mice as a function of age. J. Immunol. **148**: 1620–1629.

Kingston R., Jenkinson E.J., and Owen J.J.T. (1985). A single stems cell can recolonize and embryonic thymus, giving rise to phenotypically distinct T-cell populations. Nature **317**: 811–813.

Marshak-Rothstein A., Fink P., Grindley T., Raulet D.H., Bevan M.J., and Gefter M.L. (1979). J. Immunol. **122**: 2491–2497.

Muller-Sieburg C.E., Whitlock C.A. and Weissman I.L. (1986). Isolation of two early B lymphocyte progenitors from mouse marrow: A committed pre-pre-B cell and a clonogenic. Thy-1-low haematopoietic stem cell. Cell **44**: 653–662.

Ohteki T., Okuyama R., Seki S., Abo T., Sugiura K., Kusumi A., Ohmori T., Watanabe H. and Kumagai K. (1992). Age-dependent increase of extrathymic T cells in the liver and their appearance in the periphery of older mice. J. Immunol. **149**: 1562–1570.

Sarimiento M., Glasebrook A.L., and Fitch F.W. (1980). IgG or IgM monoclonal antibodies reactive with different determinants in the molecular complex bearing Lyt-2 antigen block T cell-mediated cytolysis in the absence of complement. J. Immunol. **125**: 2665–2672.

Scollay R., Wilson A., D'Amico A., Kelly K., Egerton M., Pearse M., Wu L. and Shortman K. (1988). Developmental status and reconstitution potential of subpopulations of murine thymocytes. Immunol. Rev. **104**: 81–120.

Spangrude G.J., Heimfeld S., and Weissman I.L. (1988). Purification and characterization of mouse hematopoietic stem cells. Science **241**: 58–62.

Spangrude G.J., and Scollay R. (1990). Differentiation of hematopoietic stem cells in irradiated mouse thymic lobes: Kinetics and phenotype of progeny. J. Immunol. **145**: 3661–3668.

Wu, L., Scollay R., Egerton M., Pearse M., Spangrude G.J., and Shortman K. (1991). CD4 expressed on earliest T-lineage cells in the adult murine thymus. Nature **349**: 71–74.

